# The preconception health status of nongravid women aged 18 to 45 years in Arima, Trinidad: a cross-sectional study

**DOI:** 10.1186/s12884-023-06017-2

**Published:** 2023-10-10

**Authors:** Ria Bala, Rohan G Maharaj, Leela Krishna Teja Boppana, Surujpal Teelucksingh

**Affiliations:** 1 Unit of Public Health and Primary Care, Department of Paraclinical Sciences, Faculty of Medical Sciences, The University of the West Indies, St Augustine Campus, Trinidad; 2 Unit of Internal Medicine, Department of Medicine, Faculty of Medical Sciences, The University of the West Indies, St Augustine Campus, Trinidad

**Keywords:** Preconception care, Screening, Maternal health, Foetal health, Trinidad

## Abstract

**Background:**

Preconception care (PCC) is the term used for activities and interventions designed to address and prevent problems related to pregnancy, the neonatal period and childhood. This study assessed maternal health status prior to conception in Trinidad by means of a screening tool, physical measurements, and laboratory samples.

**Methods:**

A cross-sectional study was conducted among women aged 18–45 years at a primary care centre in Arima, Trinidad. A *de novo* PCC screening tool was used to assess 13 domains of high-risk pregnancy in participants. These domains included dietary details, gynaecological and obstetric histories, and genetic and vaccination histories, among others. Blood pressure, weight, height, and waist circumference were recorded, and a capillary blood sample was used to determine random blood glucose and HbA1c levels. All data were coded and entered into SPSS ver. 21.

**Results:**

A total of 400 nongravid participants were recruited, of whom 366 were included in the final analysis. Most **(**96.7%) had one or more risk factors for adverse pregnancy outcomes. These included overweight (27%), obesity (35%), central obesity (69.4%), and impaired glucose tolerance/diabetes mellitus (IGT/DM) (26.2%). Additionally, a sedentary lifestyle and diet high in processed food/fats were self-reported by 74.9% and 88.8% of participants, respectively. Only 13.1% had planned to conceive, and of those who had no immediate plans to conceive, 76.4% were currently sexually active, and many (60.7%) did not use birth control techniques. More than half (57.1%) had never had a pap smear. On the other hand, 86.3% knew their HIV status. Self-reported percentages for vaccination were as follows: MMR (100%), tetanus (17.5%), hepatitis B (11.5%) and influenza (2.7%). The majority (82.8%) of participants had not visited the dentist in the past year, with 35.9% of these individuals reporting symptoms of periodontitis. Segments of the population had multiple risk factors; for example, 23.7% of participants were overweight or obese and had an elevated HbA1c level.

**Conclusions:**

Unexpectedly, most participants had a risk factor for an adverse pregnancy outcome, and many had multiple risk factors. There is a strong case for enhanced preconception care for women in Trinidad.

**Supplementary Information:**

The online version contains supplementary material available at 10.1186/s12884-023-06017-2.

## Background

Preconception care (PCC) is the term used for a wide range of activities and interventions intended to address and prevent specific problems related to pregnancy, the neonatal period and childhood. PCC can be defined as “the provision of biomedical and behavioural interventions prior to pregnancy in order to optimize women’s wellness and subsequent pregnancy outcomes with the aim to improve not only foetal, infant, and maternal health but also the health of the whole family and the future well-being of the offspring” [1]. Internationally, preconception health promotion is at the forefront of improving maternal and child health.

There is emerging evidence that “early prenatal care is too late” [2]. By the time a pregnancy is recognized, or the woman makes it to her first prenatal visit, most foetal structures are already well advanced in their development. Interventions to improve the health of mothers and children at this stage may be too late to have any effect. Many international organizations now recommend routine PCC [2, 3]. The March of Dimes recommends that “the key physician/primary care provider must take advantage of every health encounter to provide preconception care and risk reduction before and between conceptions—the time when it really can make a difference” [2]. The American Academy of Pediatrics (AAP) and the American College of Obstetricians and Gynecologists (ACOG) offer similar recommendations [3].

To support these recommendations, the World Health Organization (WHO) convened a meeting to develop a global consensus [4]. The list of activities included programs for addressing tobacco use prevention and tobacco use cessation, nutrition, vaccination, infertility, HIV testing and counselling, mental health, alcohol and drug use, intimate partner abuse, genetic counselling, and occupational health. The WHO has recommended a more integrated approach to PCC within health systems and moving away from vertical programs [4].

### Preconception health care in Trinidad and Tobago

It is a common clinical experience that antenatal health care in Trinidad and Tobago (T&T) does not commence until the pregnancy is well established and, in many cases, deep into the second trimester. Uche-Nwachi and colleagues in 2010 reported that 60.8% of antenatal patients had their initial visit after the first trimester [5]. This late-onset antenatal care may well contribute to the maternal and infant mortality rates in T&T. For example, PAHO reported that the maternal mortality rate (MMR) was 46.9 per 100,000 live births and the infant mortality ratio (IMR) was 12 per 1,000 live births in T&T in the early 2010s [6]. These rates are well above those of developed nations, where the MMR and IMR are estimated at 12 per 100,000 live births and 6 per 1,000 live births, respectively [6, 7].

Additionally, in T&T, many pregnancies are unplanned. In 2009, Ali and her colleagues reported a rate of unplanned pregnancies of 60.4% in North Central Trinidad [8]. These pregnancies are often accompanied by preexisting conditions such as sickle cell anaemia, thalassemia, and alcohol use [9]. Research suggests that drug use, mental health problems and chronic noncommunicable diseases such as obesity, diabetes, and hypertension often go unchecked and may therefore contribute to these statistics [6].

Currently, in T&T, there are existing programs that offer care to women of reproductive age, but they exist in silos, such as prenatal care, family planning clinics or well-infant checks, and do not adopt a PCC perspective. There is no formal service taking an integrated approach to identify and manage risk factors that affect maternal and foetal outcomes. As such, the current preconception health status of women of reproductive age is unknown. This study aimed to determine the prevalence of preconception health risks in a population of women aged 18–45 years in a Trinidadian primary care setting.

## Methods

### Study design

This was a cross-sectional study.

### Setting

Health care in Trinidad and Tobago may be sourced via public or private health care facilities. The public sector, governed by the Ministry of Health, comprises 5 regional health authorities (RHAs) within which there are various hospitals and 9 district health facilities. The public health care system is financed by the government and taxpayers and is available free of charge at the point of service for all citizens on a walk-in basis [10]. The study was conducted at the Arima District Health Facility from December 4th, 2016, to January 14th, 2017.

The Arima Borough is semirural, with a catchment population of 33 606 people according to the 2011 census. Females in the reproductive age group make up 20% of the population of this region. The ethnic composition of the population is distributed among 3 major groups: African, South Asian and mixed ethnicity, with the former two groups being more common. Chinese, European and Middle Eastern populations together comprise a minority [11]. RB was an attending physician at the institution at the time of the study. The centre was chosen because of its convenience and because it is the major medical institution in the district.

### Participants

Subjects were included if they were female and between the ages of 18 and 45 years. Pregnant women were not excluded. Exclusion criteria were women who had completed their families and were on contraception or those who were sterilized.

Following registration by a clerical officer, a *de novo* preconception care screening checklist (See Additional File 1) was administered by RB in English. Consecutive patients attending the walk-in service were invited to participate.

### Data sources/Measurements

The patients’ blood pressure, weight, height, and waist circumference were recorded using the WHO STEPS Protocol [12].

Measurements were repeated three times, and the average value was used. A capillary blood sample was obtained for the determination of random blood glucose and HbA1c levels. Samples were analysed via a DCA 2000^Ⓡ^ Analyser, which is very useful for point-of-care testing [13].

### Variables: the preconception care screening tool

To ensure face and content validity, the preconception care screening tool was modelled after the recommendations published by the WHO in 2013 [14]. RB created the first version, and RGM and ST reviewed and edited the instrument for ease of use and item flow. The following domains were assessed [15–17]:

1. Intent of Pregnancy.
2. Gynaecological History.
3. Obstetric History.
4. Medical History.
5. Genetic History.
6. Immunization History.
7. Medication History.
8. Diet and Exercise.
9. Lifestyle.
10. Environmental Health.
11. Emotional Health.
12. Dental Health.
13. Partner’s History.

See Additional File 1 for an in-depth description of these domains and the survey instrument. The screening tool took the format of a checklist to aid in efficient patient screening. If a risk to maternal health was identified, steps to cue further management based upon the current guidelines were included on the form. In this study, impaired glucose tolerance (IGT) or prediabetes was defined by an HbA1c range of 5.7 to 6.5%, with diabetes (DM) being defined at an HbA1c level ≥ 6.5% [18, 19].

The Indo-Caribbean (originally from South Asia) population has a higher percentage of body fat than the Caucasian population of the same age, sex and BMI; therefore, in their expert consultation in 2004, the World Health Organization (WHO) highlighted the need for public health action to be taken in this subgroup at a lower cut-off limit [20]. For Indo-Caribbean people, the BMI categories suggested for public health actions are as follows: less than 18.5 kg/m^2^ (underweight); 18.5–23 kg/m^2^ (normal); 23–27.5 kg/m^2^ (overweight); and 27.5 kg/m^2^ or higher (obesity).

All data were coded and stored in a password-protected computer accessed solely by RB.

### Bias

The main biases in a survey include sampling, nonresponse, question order, recall and desirability biases. In our limitations, we acknowledge that the final sample was not entirely representative of the overall Trinidad and Tobago population. Patients were invited by the administrative staff immediately upon presentation at the clinic. This could affect the willingness of those invited to favour participation in the study. It was stressed in the consenting process, however, that refusal would not affect their subsequent care. No special techniques were taken to reduce recall or desirability biases.

### Study size determination

Sample size was determined using a margin of error of 5% and a confidence level of 95%. The following equation for estimating a population proportion with specified absolute precision, which is based on simple random sampling, was used to calculate the sample size:

$$n=[(Z2\alpha/2)(P(1-P))]/d2.$$

where “n” is the calculated sample size, “Z” is the critical value for a 95% confidence level (1.96), “P” is the anticipated population proportion and “d” is the margin of error. When “P” was unknown, the safest choice for the anticipated population proportion of 50% was used, and a sample size of 364 patients was calculated. Applying a 10% nonresponse rate gave a final sample size of 400 patients.

### Ethics approval

Approvals for conducting this study were provided by the Institutional Review Boards of The University of the West Indies (UWI), St. Augustine, Trinidad and the NCRHA, Trinidad. An informed consent form was obtained from each patient, which was signed in the presence of a witness. The patient’s autonomy was respected; they were informed that they could discontinue the study at any time without affecting the care they would receive, and their participation was not coerced. Confidentiality was always maintained. The PCC screening tool did not gather any identification data. After the data were entered into statistical software and analysed, all physical records were destroyed.

### Statistical methods

SPSS ver. 21.0 was used to construct various frequency tables. Demographic variables were described by standard subgroups, such as male and female, age, reported ethnicity, completed examination, parity and the presence of diabetes mellitus and hypertension, as well as by numbers and proportions. No analytic methods are presented. A sensitivity analysis was conducted to examine the rates of overweight and obesity in the Indo-Trinidadian population if the adjusted BMI cut-off points were applied. Estimates were also calculated for participants with multiple risk factors, such as being overweight and obese and having an elevated HbA1c level.

## Results

A total of 400 consecutive subjects were invited to participate. There were no refusals. Thirty-four patients were excluded because they had completed their families or had undergone sterilization.

The majority (205; 56%) of participants were aged between 18 and 29 years, and 6.5% were over 40 years of age. Most (56.0%) self-identified as Afro-Trinidadian, followed by mixed ethnicity (26.8%) and Indo-Trinidadian (17.2%). More than half of the participants were multiparous (57.9%), and none were currently pregnant. Most (83.6%) had attained a secondary or higher level of completed education. Approximately 70% of participants had a positive family history (FH) of diabetes, and 54% had a FH of hypertension (see Table 1).

**Table 1:** Demographic characteristics of a female population aged 18–45 years before pregnancy in Trinidad in 2016-17

		Age Group/years					Total
		18–24	25–29	30–34	35–39	40–45	18–45
		N (%) 116 (100)	N (%) 89 (100)	N (%) 71 (100)	N (%) 66 (100)	N (%) 24 (100)	N (%) 366 (100)
Single or Partnered	Single	50 (43.1)	34 (38.2)	27 (38)	21 (31.8)	10 (41.7)	142 (38.7)
	Partnered	66 (56.9)	55 (61.8)	44 (62)	45 (61.8)	14 (58.3)	224 (60.6)
Marital Status	Partnered	63 (95.5)	41 (74.5)	21 (47.7)	20 (44.4)	7 (50.0)	152 (67.8)
	Married	3 (4.5)	14 (25.5)	23 (52.3)	25 (55.6)	7 (50.0)	72 (32.2)
	Divorced	0 (0)	0 (0)	0 (0)	0 (0)	0 (0)	0 (0)
	Other	0 (0)	0 (0)	0 (0)	0 (0)	0 (0)	0 (0)
Ethnicity	Afro-Trinidadian	61 (52.6)	49 (55.1)	41 (57.7)	36 (54.5)	18 (75.0)	205 (56.0)
	Indo-Trinidadian	20 (17.2)	19 (21.3)	10 (14.1)	12 (18.2)	2 (8.3)	63 (17.2)
	Mixed	35 (30.2)	21 (23.6)	20 (28.2)	18 (27.3)	4 (16.7)	98 (26.8)
Highest level of completed education	None	2 (1.7)	1 (1.1)	1 (1.4)	0 (0)	0 (0)	4 (1.1)
	Primary Level	19 (16.4)	9 (10.1)	13 (18.3)	8 (12.1)	7 (29.2)	56 (15.3)
	Secondary Level	67 (57.8)	55 (61.8)	46 (64.8)	51 (77.3)	14 (58.3)	233 (63.7)
	Tertiary Level	28 (24.1)	24 (30.0)	11 (15.5)	7 (10.6)	3 (12.5)	73 (19.9)
Parity	0	96 (82.8)	40 (44.9)	12 (16.9)	9 (13.6)	1(4.2)	158 (43.2)
	1–2	17 (14.7)	41 (46.1)	41 (57.7)	36 (54.5)	11 (45.8)	146 (39.9)
	3+	3 (2.6)	8 (9.0)	18 (25.4)	21 (31.8)	12 (50.0)	66 (16.9)
Current Frequency of Sexual Intercourse (times/wk)	0	26 (22.4)	22 (24.7)	11 (15.5)	11 (16.7)	8 (33.3)	78 (21.3)
	< 1	49 (42.2)	29 (32.6)	27 (38.0)	24 (36.4)	9 (27.3)	138 (37.7)
	2–3	30 (25.9)	33 (37.1)	27 (38.0)	25 (37.9)	5 (20.8)	120 (32.7)
	> 3	11 (9.5)	5 (5.6)	6 (8.5)	6 (9.1)	2 (8.3)	30 (8.7)
Family History of Diabetes Mellitus	Yes	72 (62.1)	61 (68.5)	51 (71.8)	49 (74.2)	22 (91.7)	255 (69.7)
	No	44 (37.9)	28 (31.5)	20 (28.2)	17 (25.8)	2 (8.3)	111 (30.3)
Family History of Hypertension	Yes	50 (43.1)	49 (55.1)	40 (56.3)	43 (65.2)	16 (75.0)	198 (54.1)
	No	66 (56.9)	40 (44.9)	31 (43.7)	23 (34.8)	8 (25.0)	168 (49.5)

The clinical characteristics of the sample population are shown in Table 2. Body mass index (BMI) was in the normal range (BMI 18.5–25 kg/m^2^) in only 38% of patients, while 27% were overweight (BMI 25–30 kg/m^2^) and 35% were obese (BMI ≥ 30). Of the 366 patients, 69.4% were assessed as having central obesity with a waist circumference ≥ 80 cm. More than one-third (35.5%) exhibited *acanthosis nigricans*—a marker of insulin resistance. In terms of glycaemic status, 73.8% were normoglycaemic (HbA1c level < 5.7%), 21.6% were prediabetic/had impaired glucose tolerance (HbA1c level = 5.7–6.5%) and 4.6% fulfilled the criteria for diabetes (HbA1c level > 6.5%). While only 6% reported a personal history of hypertension, more than half of the subjects had an elevated blood pressure either in the pre- or hypertensive range [21]. When BMI was calculated for the Indo-Trinidadian population using the recommended cut-off points [20], the combined rate of overweight and obesity increased from 50 to 70%.

**Table 2:** Clinical characteristics of a female population aged 18–45 years before pregnancy in Trinidad in 2016-17

		Age Group/years					Total
		18–24	25–29	30–34	35–39	40–45	18–45
		N (%) 116 (100)	N (%) 89 (100)	N (%) 71 (100)	N (%) 66 (100)	N (%) 24 (100)	N (%) 366 (100)
BMI Overweight	< 25	63 (54.3)	38 (42.7)	24 (33.8)	11 (16.7)	3 (12.5)	139 (38)
	> 25	53 (45.7)	51 (57.3)	47 (66.2)	55 (83.3)	24 87.5)	227 (62)
Waist Circumference Range	< 80 cm	57 (49.1)	26 (29.2)	16 (22.5)	11 (16.7)	2 (8.3)	112 (100)
	≥ 80 cm	59 (50.9)	63 (70.8)	55 (77.5)	55 (83.3)	22 (91.7)	254 (100)
Presence of *Acanthosis Nigricans*	Yes	22 (19.0)	33 (37.1)	27 (38.0)	31 (47.0)	17 (70.8)	130 (35.5)
	No	94 (81.0)	56 (62.9)	44 (62.0)	35 (53.0)	7 (29.2)	236 (64.5)
Self-reported Hypertension	No	109 (94.0)	78 (87.6)	59 (83.1)	46 (69.7)	17 (70.8)	344 (94.0)
	Yes	7 (6.0)	11 (12.4)	12 (16.9)	20 (30.3)	7 (29.2)	22 (6.0)
Estimated BP Classification	Normo-tensive	70 (60.3)	44 (49.4)	31 (43.7)	14 (21.2)	7 (29.2)	116 (45.4)
	Prehypertension	39 (33.6)	34 (38.2)	28 (39.4)	32 (48.5)	10 (41.7)	144 (39.3)
	Stage 1 Hypertension	6 (5.2)	10 (11.2)	10 (14.1)	15 (22.7)	6 (25.0)	46 (12.6)
	Stage 2 Hypertension	1 (0.9)	1 (1.1)	2 (2.8)	5 (7.6)	1 (4.2)	10 (2.7)
Glycaemic status	HbA1c < 5.7%	105 (90.5)	66 (74.2)	54 (76.1)	37 (56.0)	8 (33.3)	270 (100)
	HbA1c ≥ 5.7–6.5%	10 (8.6)	21 (23.6)	14 (19.7)	24 (36.4)	10 (41.7)	79 (100)
	HbA1c ≥ 6.5%	1 (0.9)	2 (2.2)	3 (4.2)	5 (7.6)	6 (25.0)	17 (100)

Table 3 shows the lifestyle measures. A total of 8.2% of participants had a history of tobacco use, 25.1% reported exposure to second-hand tobacco smoke, 30.9% consumed alcohol, and 74.9% reported a sedentary lifestyle with no exercise. Current sexual activity was reported by 78.7% of participants. Most (86.9%) had no plans for an immediate pregnancy, yet 60.7% were not utilizing any contraceptive technique.

**Table 3:** Lifestyle characteristics of a female population aged 18–45 years before pregnancy in Trinidad in 2016-17

		Age Group					TOTAL
		18–24	25–29	30–34	35–39	40–45	18–45
		N (%) 116 (100)	N (%) 89 (100)	N (%) 71 (100)	N (%) 66 (100)	N (%) 24 (100)	N (%) 366 (100)
Currently exercises	Yes	30 (25.9)	23 (25.8)	22 (31.0)	13 (19.7)	4 (16.7)	92 (25.1)
	No	86 (74.1)	66 (74.2)	49 (69.0)	53 (80.3)	20 (83.3)	274 (74.9)
Currently smokes cigarettes/tobacco products	Yes	8 (6.9)	7 (7.9)	5 (7.0)	9 (13.6)	1 (4.2)	30 (8.2)
	No	108 (93.1)	82 (92.1)	66 (93)	57 (86.4)	23 (95.8)	336 (91.8)
Current exposure to secondhand smoke	Yes	29 (25.0)	23 (25.8)	15 (21.1)	17 (25.8)	8 (33.3)	92 (25.1)
	No	87 (75.0)	66 (74.2)	56 (78.9)	49 (74.2)	16 (66.7)	274 (74.9)
Any current alcohol consumption	Yes	32 (27.6)	26 (29.2)	27 (38.0)	20 (30.3)	8 (33.3)	113 (30.9)
	No	84 (72.4)	63 (70.8)	44 (62.0)	46 (69.7)	16 (66.7)	253 (69.1)
Planning to get pregnant in 6 months	Yes	10 (8.6)	10 (11.2)	13 (18.3)	12 (18.2)	3 (12.5)	48 (13.1)
	No	106 (91.4)	79 (88.8)	58 (81.7)	54 (81.8)	21 (87.5)	318 (86.9)
Self-reported High-Fat Diet	Yes	80 (70.0)	56 (62.9)	37 (52.1)	29 (43.9)	15 (62.5)	217 (59.3)
	No	36 (30.0)	33 (37.1)	34 (47.9)	37 (56.1)	9 (37.5)	149 (40.7)
Self-reported High Processed Food Diet	Yes	78 (67.2)	49 (55.1)	36 (50.7)	31 (47.0)	10 (41.7)	204 (55.7)
	No	38 (32.8)	40 (44.9)	35 (49.3)	35 (53.0)	14 (58.3)	162 (44.3)
Alcohol History of the Partner	Yes	33 (51.6)	26 (46.4)	20 (47.6)	23 (51.1)	5 (35.7)	107 (48.4)
	No	31 (48.4)	30 (53.6)	22 (52.4)	22 (48.9)	9 (64.3)	114 (51.2)

Table 4 highlights the frequency of contraception, immunization, and cervical cancer screening. Among 130 subjects who were utilizing contraception, a barrier method with male condoms was the most common (52%), followed by oral contraceptive pills (18%), IUDs (12%) and depot preparations (< 1%). More than half (57.1%) had never had a pap smear. Vaccination rates for MMR, tetanus, hepatitis B, varicella zoster and annual influenza vaccines were reported as follows: 100%, 17.5%, 11.5%, 4.6% and 2.7%, respectively. Most (82.8%) participants had not seen a dentist within the last year, and 35.9% of these individuals had symptoms of periodontitis.

**Table 4:** Preventive measures practiced among a female population aged 18–45 years before pregnancy in Trinidad in 2016-17

		Age Group					TOTAL
		18–24	25–29	30–34	35–39	40–45	18–45
		N (%) 116 (100)	N (%) 89 (100)	N (%) 71 (100)	N (%) 66 (100)	N (%) 24 (100)	N (%) 366 (100)
Birth Control Usage	Yes	45 (38.8)	34 (38.2)	23 (32.4)	22 (33.3)	9 (37.5)	130 (35.5)
	No	71 (61.2)	55 (61.8)	48 (61.6)	44 (66.7)	15 (62.5)	236 (64.5)
Type of Birth Control	Oral Contraceptive Pills	5 (11.1)	6 (17.6)	5 (22.7)	5 (22.7)	1 (11.1)	22 (17.3)
	Depot Preparations	3 (6.7)	5 (14.7)	5 (22.7)	0 (0.0)	0 (0.0)	13 (9.8)
	IUDs	0 (0.0)	3 (8.8)	3 (13.6)	6 (27.3)	4 (36.4)	16 (12.0)
	Condoms	32 (71.1)	15 (44.1)	7 (31.8)	10 (45.5)	3 (33.3)	67 (50.4)
	Other	5 (11.1)	5 (14.7)	2 (9.1)	1 (4.5)	1 (11.1)	14 (10.8)
Pap Smear Done within the Past 3 Years	Yes	8 (6.9)	29 (32.6)	33 (46.5)	31 (47.0)	10 (41.7)	111 (30.3)
	No	3 (2.6)	9 (10.1)	11 (15.5)	18 (27.3)	5 (20.8)	46 (12.6)
	Never	105 (90.5)	51 (57.3)	27 (38.0)	17 (25.8)	9 (37.5)	209 (57.1)
Tested for HIV	Yes	79 (68.1)	81 (91.0)	69 (97.2)	65 (98.5)	24 (100.0)	318 (86.9)
	No	37 (31.9)	8(9.0)	2 (2.8)	1 (1.5)	0 (0.0)	48 (13.1)
Dental Visit within the Past 6 Months	Yes	11 (18.6)	7 (15.2)	4 (11.8)	10 (23.8)	1 (9.1)	33 (17.2)
	No	48 (81.4)	39 (84.8)	30 (88.2)	32 (76.2)	10 (90.9)	159 (82.8)
One or More Symptoms of Periodontitis	Yes	18 (30.5)	17 (40.0)	15 (44.1)	16 (38.1)	3 (27.3)	69 (35.9)
	No	41 (69.5)	29 (60.0)	19 (55.9)	26 (61.9)	8 (72.7)	123 (64.1)
MMR Vaccine	Yes	116 (100)	89 (100)	71 (100)	66 (100)	24 (100)	366 (100)
	No	0 (0)	0 (0)	0 (0)	0 (0)	0(0)	0 (0)
Hepatitis B Vaccine	Yes	13 (11.2)	13 (14.6)	8 (11.3)	6 (9.1)	2 (8.3)	42 (11.5)
	No	103 (88.8)	76 (85.4)	63 (88.7)	60 (90.9)	22 (91.7)	324 (88.5)
Varicella Zoster Vaccine	Yes	5 (4.3)	4 (4.5)	5 (7.0)	1 (1.5)	2 (8.3)	17 (4.6)
	No	111 (95.7)	85 (95.5)	66 (93.0)	65 (98.5)	22 (91.7)	349 (95.4)
Tetanus Vaccine	Yes	21 (18.1)	16 (20.0)	16 (22.5)	7 (10.6)	4 (16.7)	64 (17.5)
	No	95 (81.9)	73 (80.0)	55 (77.5)	59 (89.4)	20 (83.3)	302 (82.5)
Influenza Vaccine	Yes	2 (1.7)	2 (2.2)	2 (2.8)	3 (4.5)	1 (4.2)	10 (2.7)
	No	114 (98.3)	87 (97.8)	69 (97.2)	63 (95.5)	23 (95.8)	356 (97.3)

Table 5 illustrates participant subgroups with multiple risk factors: 23.7% of participants were overweight or obese and had an elevated HbA1c level; 7.4% were overweight or obese and had an elevated HbA1c level and stage 1 or 2 hypertension.

**Table 5:** Multiple risk factors among a female population aged 18–45 years before pregnancy in Trinidad in 2016-17

Multiple risk factors	N (%)
Overweight or Obese with an elevated HbA1c level > 5.7%	87 (23.7)
Overweight or Obese with an elevated HbA1c level > 5.7% AND Stage 1 or Stage 2 Hypertension	27 (7.4)
Overweight or Obese with an elevated HbA1c level > 5.7% AND Stage 1 or Stage 2 Hypertension AND Alcohol use	11 (3.0)

## Discussion

This study provided baseline statistics on preconception factors that can contribute to complications during pregnancy. Unexpectedly, most (96.7%) women aged 18–45 years in this Trinidadian primary care population had at least one risk factor for adverse pregnancy outcomes. Several participants had multiple risk factors, as illustrated in Table 5.

### Overweight/obesity

Whether categorized by BMI or waist circumference, our results indicate that obesity is highly prevalent in the demographic under study. Maternal obesity is strongly associated with foetal macrosomia [22]. Furthermore, macrosomia is associated with a multitude of poor foetal outcomes, including musculoskeletal injuries such as shoulder dystocia and brachial plexus injury, respiratory complications including meconium aspiration and perinatal asphyxia, hypoglycaemia and foetal demise [23]. In addition, there is an association between foetal macrosomia and long-term health consequences in childhood as well as adulthood, such as obesity, diabetes, and heart disease [24–26]. A large proportion of the sample admitted to having a poor diet (88%) and not exercising (74.9%).

The recommended cut-off points for BMI in the Indo-Trinidadian population are lower than those for the other ethnic groups [20]. When we applied these cut-off points, the combined rate of overweight and obesity increased from 50 to 70%. This is a noteworthy increase, yet little attention and promotion are paid to these recommendations locally.

### Impaired glucose tolerance and diabetes

More than two-thirds of participants indicated a positive family history of diabetes mellitus. Approximately one-third exhibited *acanthosis nigricans*—a marker of insulin resistance. Based on the participants’ glycaemic status, a total of 26% were prediabetic, had impaired glucose tolerance (HbA1c level 5.7–6.5%) or fulfilled the criteria for diabetes (HbA1c level > 6.5%). In contrast, internationally, the prevalence of gestational diabetes (GDM) varies between 1% and 14% in all pregnant women depending on the genetic characteristics of the population and the population prevalence of type 2 diabetes mellitus, the environment of the population under study and the tools used for screening and diagnosis [27].

GDM is associated with an elevated risk of maternal and perinatal mortality, obstructed labour, spontaneous abortion, congenital abnormalities and macrosomia [28]. There is evidence to show that the increasing prevalence of obesity globally, as discussed above, directly contributes to the increasing prevalence of impaired glucose tolerance and diabetes [29–31].

### Substance use and the reproductive population

The detrimental effects of alcohol use, tobacco use and the use of other toxic substances during pregnancy are widely documented [32]. This includes foetal alcohol spectrum disorder (FASD), in which infants are at risk of preterm birth, low birth weight, abnormal facial features, and small head size [33–35].

After preconception counselling, it is possible for women to change their behaviour to reduce adverse outcomes during and after pregnancy, including reducing their alcohol and tobacco use [36]. For example, in one study after PCC counselling, significantly more women started using folic acid before pregnancy and reduced their alcohol use during the first three months of pregnancy [36]. This current study identified high levels of alcohol use and exposure to second-hand tobacco smoke before pregnancy among the female population. Given the low public health information available at the local level and the high levels of both alcohol and tobacco use by household members and the population under study, this finding is not unexpected.

### Unplanned, unwanted pregnancies and the reproductive population

In this study, 86.9% of the sample did not plan to become pregnant in the near future; however, only 39.3% used contraception. This translates to a significant proportion of patients at risk for an unplanned and/or unwanted pregnancy. Available evidence shows that unplanned pregnancies can have a negative effect on women’s lives. Many women with unplanned and unwanted pregnancies may decide to have an abortion and are thus exposed to resulting social and medical complications. Additionally, women in this situation who give birth may have an increased risk of obstetric complications [37, 38]. There is therefore a compelling argument to strengthen current health education, skill building, counselling, and advocacy for long-acting methods of contraception.

Notably, short-acting contraception was the contraceptive method of choice in 78% of the sample, with barrier protection being utilized by 50% of those using contraception. Barrier protection plays a significant role in reducing the transmission of sexually transmitted infections and should be encouraged, especially for women who are at high risk. Preconception behavioural interventions significantly reduce reinfection or new STI rates by 35% [39].

### Cervical cancer screening and the reproductive population

Cervical cancer is the second most common female cancer in the developing world, accounting for 84% of new cases worldwide (445,000 new cases in 2012) [40]. Despite the importance of having routine pap smears, 52.5% of our sample had never had a pap smear, and 13.9% had not had a recent pap smear. Enhanced PCC could improve health education about cervical screening.

### Immunization status and the reproductive population

Less than one-fifth of the participants reported being vaccinated against tetanus in the past ten years, and 2.7% were vaccinated against influenza; however, 100% had received childhood MMR vaccines. Regarding the tetanus vaccination rate, this is most likely a case of underreporting since tetanus vaccination is given as part of the DPT vaccine to all primary school students as a compulsory prerequisite for attendance. It is likely that participants were unaware what the DPT acronym stands for. Vaccination against tetanus and rubella, among others, averts a significant number of neonatal deaths and diseases [41]. The results of this study suggest inadequate vaccine coverage for influenza and hepatitis B, which are recommended internationally for all women of reproductive age [42]. Opportunistic preconception screening would allow patients’ immunization status to be updated in adulthood, prior to pregnancy.

### Periodontal disease and the reproductive population

Periodontitis has been linked to preterm labour and adverse pregnancy outcomes [43]. This study shows that more than one-third of the sample had one or more symptoms of periodontitis, and less than one-fifth had a dental visit in the past year. Therefore, there are opportunities to invest in dental health as a PCC intervention.

### Limitations

There are several limitations to the study. First, this study was conducted in East Trinidad, where 56% of the sample was of Afro-Trinidadian descent and 16.9% was of Indo-Trinidadian descent. This would affect the generalizability to the wider national population, which consists of individuals of African descent (34.2%) and of Indo-Trinidadian descent (35.2%). Additionally, this study was conducted in a public health centre that provides services to individuals with lower to middle socioeconomic status. Moreover, several of the planned tests could not be completed because of a lack of funding. These included routine blood tests such as CBC, sickle cell, HIV and VDRL tests. Had these tests been done, the burden of risk might have been found to be higher.

Nevertheless, there are several strengths. The sample size was adequate. Additionally, this was the first such study in the English-speaking Caribbean population. Importantly, we were unaware as to how poorly T&T was doing in this area, and this study can help shape local policy.

### What’s next?

These results suggest that there are real opportunities to improve the health of potential mothers and their foetuses in T&T through public health messages, physician education and the reorientation of services. A recent narrative review published in the *Lancet* established that “there is sufficient evidence from human and animal research showing that the periconceptional period is a key window during which poor maternal and paternal physiology, body composition, metabolism, and diet can induce increased risk of chronic disease in offspring—a lifetime legacy and major driver of the health burden in the 21st century” [44]. Others suggest that an integrated rather than a silo approach to preconception health promotion might be the most effective and efficient method [45–49]. Community physicians are strategically positioned to improve pregnancy-related outcomes through prenatal interventions and thus should be the foundation of such care [49]. A 2022 systematic review, while bemoaning the paucity of research, suggests that there can be favourable outcomes regarding social behaviours during pregnancy, disease activity and pregnancy outcomes. The authors conclude that the findings support the routine inclusion of PCC in preparation for pregnancy [50].

Despite overwhelming evidence that PCC has the potential to enhance the health and overall well-being of women, infants, and children globally, insufficient attention is given to the clinical reality of PCC research, service, and delivery, especially in low- and middle-income countries. The transfer of PCC guidelines from broad policy to on-the-ground practice requires a more detailed consideration of the practicalities of PCC implementation, especially within developing countries [48, 51].

## Conclusions

This study unexpectedly revealed that as many as 97% of 18–45-year-old women in Trinidadian primary care practices present with factors that increase the chances of adverse pregnancy outcomes. Many women have multiple risk factors. In Small Island Developing States (SIDS) such as Trinidad, where our human resources are an important investment, these data suggest that there is much more that we can do to protect mothers and infants. There are opportunities for further research, the broadening of public health policy and reorientation of the health system.

### Electronic supplementary material

Supplementary Material 1

## Data Availability

The *de novo* questionnaire is provided in Additional File 1. The datasets used during the current study are not available to the public, as additional analysis is ongoing. It is, however, available from the corresponding author upon reasonable request.

## List of abbreviations

BMI: Body Mass Index
CBC: Complete Blood Count
DM: Diabetes Mellitus
DPT: Diphtheria, Pertussis and Tetanus
GDM: Gestational Diabetes Mellitus
HbA1c: Glycosylated Haemoglobin
HIV: Human Immunodeficiency Virus
IGT: Impaired Glucose Tolerance
IMR: Infant Mortality Rate
MMR: Maternal Mortality Rate
MMR: Measles, Mumps and Rubella
NCD: Noncommunicable Disease
NCRHA: North Central Regional Health Authority
PAHO: Pan American Health Organization
PAP: Papanicolaou
PCC: Preconception Care
SCD: Sickle Cell Disease
SD: Standard Deviation
SPSS: Statistical Package for Social Sciences
STEPS: STEPwise Approach to NCD Risk Factor Surveillance
T&T: Trinidad and Tobago
UWI: The University of the West Indies
VDRL: Venereal Disease Research Laboratory
WC: Waist Circumference
WHO: World Health Organization
