# Supplementary material for: The preconception health status of nongravid women aged 18 to 45 years in Arima, Trinidad: a cross-sectional study
Source: BMC Pregnancy Childbirth. 2023 Oct 10;23:720. doi: 10.1186/s12884-023-06017-2 (PMC10566166; doi:10.1186/s12884-023-06017-2)
Supplement: Supplementary file 1 — Supplementary Material 1 [file 12884_2023_6017_MOESM1_ESM.docx]

**ADDITIONAL FILE 1**

**Ethics approval and consent to participate**: Approvals for conducting this study were provided by the Institutional Review Boards of The University of the West Indies, St. Augustine, Trinidad (CEC093/12/15) and the North Central Regional Health Authority, Trinidad. The approved protocols were carried out in strict adherence to the principles enunciated in the Declaration of Helsinki. All questionnaire respondents were informed of the study purpose; they all signed the consent forms after it was explained to them and agreed to respond to the questionnaire. This was completed voluntarily and anonymously. The IRB approval letter can be found at the end of this document.

**Description of the Preconception Care Screening Tool**

The preconception care screening tool was modelled after the recommendations by the WHO in 2013 on areas to be targeted in the preconception care package, to ensure content validity of the screening tool [15]. It aims to holistically screen the patient for any risk factors for adverse perinatal outcomes and implement a multidisciplinary approach to reduction of these identified factors. It is divided into various domains:

1. Intent of Pregnancy – these questions aim to ensure that females in the reproductive age group are having planned pregnancies. Those that have no intention of getting pregnant are advised to utilize their preference of contraception [18, 19]. Those that wish to get pregnant, frequency and timing of intercourse are assessed to increase chances of conception as well as the initiation of folic acid (400µg) supplementation for reduction of neural tube defects and other birth defects [20, 21]. The opportunity is also taken to identify any potential risks to future pregnancy and take the necessary steps to reduce these risks.
2. Gynecological History – it is important to ensure that females are having regular menses (regular cycles and flow) which indicate normal ovulatory cycles. If patients are having irregular menses or menorrhagia they should be subsequently screened for uterine fibroids, polycystic ovarian syndrome or referred appropriately. Routine pap smears are proven to reduce the mortality associated with cervical cancer, it is important that the public is educated about the importance of this and referred for routine testing [22]. Any surgical procedures to the uterus or cervix that may affect pregnancy are identified and advice subsequently given. All patients are screened and treated for any potential sexually transmitted infections; barrier contraception is recommended for those at high risk [23-26].
3. Obstetric History – Any prior obstetric history that places the patient into a high-risk pregnancy is identified (previous spontaneous miscarriages, fetal macrosomia, multigravidarum, pre-eclampsia, gestational diabetes, etc.) and patient would be referred at an early stage for specialist care.
4. Medical History - chronic medical conditions are identified, steps are taken to ensure control of the condition and if necessary, referral for specialist intervention [27]. Patients with diabetes mellitus are counselled about the importance of glycemic control and achieving target HbA1c level to reduce the risk of congenital abnormalities [28-30].
5. Genetic History – patients are screened for personal, partner or family history of congenital anomalies or genetic disorders. They are referred for genetic counseling when risk factors are identified and provide carrier testing when appropriate to determine risk to future pregnancy [31].
6. Immunization History – patient’s immunization status is identified and referred to be vaccinated as required. The aim is to update hepatitis B; influenza; measles, mumps, rubella; Tdap; and varicella immunizations as needed in patients who wish to become pregnant [32].
7. Medication History – Patients are screened for use of teratogenic medications and are changed to safer medications if possible. The fewest medications at the lowest dosages needed to control disease are recommended [33].
8. Diet and Exercise – Patient’s BMI is assessed and those that are overweight, obese, or underweight are counselled on achieving a healthy body weight before becoming pregnant. These patients are referred to a dietician and started on an exercise prescription as necessary [34]. Patients are also counselled on limiting mercury exposure and avoiding food-borne illnesses [35]. A patient’s caffeine intake is analyzed and they are counselled on reduction/cessation if they are trying to conceive [36].
9. Lifestyle – patients who are identified as smokers are referred to a smoking cessation program and counselled on the harmful effects of smoking. Patients’ alcohol/recreational drug use is assessed and reduction/cessation in pregnancy is advised. Any sign of dependence prompts brief behavioral interventions to reduce tobacco, alcohol, and drug use as well as referral to the appropriate clinic as necessary.
10. Environmental Health – any environmental factor that poses a teratogenic threat is identified and the patient is educated about avoidance during conception and pregnancy, including lead, mercury, radiation, pesticides, and cat/rodent feces [37].
11. Emotional Health – Patients are screened for signs of intimate partner violence and depression. Any positive screened patient is referred appropriately for further support, after safety is assessed [38-39].
12. Dental Health – Patients are screened for symptoms of periodontitis. Any patient who has not had a routine dental checkup or exhibits signs of periodontal disease is referred for further dental care [40].
13. Partner’s History – Patients’ partners’ genetic history are determined. All risks to conception are identified and reduction advised, including smoking, alcohol and drug use [41].

The screening tool took the format of a checklist to aid in efficient screening of the patient. If a risk to maternal health was identified, steps for further management of the patient are recommended on the form. These recommendations were based on recent clinical guidelines. The physician was able to assess if the patient adequately satisfies the above domains and is therefore at a low risk for subsequent pregnancy.

**APPENDIX II**

**Preconception Counselling Screening Tool**

Marital Status: □ Single □Partnered □Married □Divorced □Other

Study Number: Date of Birth:

Contact Number(s): Age:

Address:

Demographics:

Race: □ Afro Trinidadian □ Indo Trinidadian □ Mixed

Highest level of education obtained: □ None □ Primary School □ Secondary School □ Tertiary Level

| Intent of Pregnancy: | Are you planning to get pregnant in the next 6 months? □ YES □ NO  In the next 12 months? □ YES □ NO  Is your partner aware and supports your pregnancy plan? □ YES □ NO  How often are you currently having sexual intercourse? (times/week) □ 0 □ ≤1 □ 2-3 □ >3  Are you using currently birth control methods? □ YES □ NO  If yes, what type? □ Oral (pills) □ Depo □IUD □ Condoms □ Other | □ Advised on contraception  □ Advised on optimal frequency of intercourse for conception  □ Referred to gynecologist/ fertility specialist |
| --- | --- | --- |
| Gynecological History: | LMP: □ <1/12 □> 1/12 Regular cycles? □ YES □ NO  How many sanitary products do you use a day?  □ 1-7 □ 7-16 □ >16  Have you had a pap smear in the past 3 years? □YES □ NO □ Never  What were the results? □ Normal □ Abnormal  Have you had surgery on your uterus, ovaries, tubes, LEEP? □ YES □ NO  Do you have a history or have ever been treated for any of the following STI’s? □ YES □ NO If yes:  □ Genital Herpes □ HPV □ Genital Warts □ Chlamydia □ Syphilis □ Gonorrhea  □ Trichomonas □ Yes, unknown  Have you ever been tested for HIV? □ YES □ NO  What were the results? □ POSITIVE □ NEGATIVE | □ Advised on pap smear  □ High risk of cervical incompetence  □ Advised on barrier contraception  □ Referred for screening for STI’s |
| Obstetric History: | Have you ever been pregnant? □ YES □ NO  Gravida Parity  Highest birth weight/kg:  Any: □ Prior ectopic/s □ Prior fetal deaths □Congenital abnormalities  □ Prior preterm births  Any complications during previous pregnancy?  □ Pre-eclampsia □ GDM □ Anemia □ APH/PPH | □ Counselled on risk recurrence of complications  □ Referred to Specialist care |
| Medical History:  Do you have a history of?  Does your partner have a history of? | □ Diabetes □ Seizures □ Thyroid Disease □ Asthma □ Anemia  □ Hepatitis □ Hypertension □ Sickle cell disease □ Depression □ Other ____________  □ SCD □ HIV □ Hepatitis □STI | □ Counselled on control of condition/risk to pregnancy/ need for planned pregnancy  □ Referred to Specialist care |
| Genetic History:  Do you or your partner have a family history of? | □ SCD □ Downs Syndrome □ Birth defects □ Diabetes □ Other _________ | □ Genetic counselling  □ Referral |
| Immunization History: | □ MMR □ Hepatitis B □ Varicella Zoster □ Tetanus  □ Influenza | □ Vaccination to be updated |
| Medication History: | Are you taking any of the following? □ Folic acid □ Calcium □ Iron  □ Diet pills □ Herbal remedies □ Over the counter medication  Are you taking any medications? □ YES □ NO  If yes list: _______________  Are you allergic to any medications? □ YES □ NO  If Yes list: ________________ | □ Start folic acid 400µg/day  □ Discontinue herbal medication, medication harmful in pregnancy |
| Diet and Exercise: | Weight/kg: Waist Circumference/inches:  Height/m:  Do you eat three meals a day? □ YES □ NO  Do you eat fruit and vegetables every day? □ YES □ NO  Do you consume raw meat or fish? □ YES □ NO  Do you follow a special diet? (vegetarian, low salt etc.) □ YES □ NO  Is your diet high in fats? □ YES □ No  Is your diet high in processed foods? □ YES □ NO  How many glasses of the following do you drink every day?  Tea Water  Coffee Milk  Cola Other  Do you exercise? □ YES □ NO  How many minutes per day/days per week?  □ <120 □ 120-150 □>150  Intensity: □ Mild □Moderate □ Vigorous | □ Advised healthy diet  □ Referred to dietician  □ Advised on appropriate exercise  □ Advised to limit caffeine consumption |
| Lifestyle: | Do you smoke cigarettes or use tobacco products? □ YES □ NO  If so, how many pack years? _____________  Are you exposed to secondhand smoke? □ YES □ NO  Do you drink alcohol? □ YES □ NO  If so, how much, how often: unit/d_____________  Do you or have you used drugs?  □ Cocaine □ Marijuana □ No □ Other __________  Do you use saunas or hot tubs? | □ Counselled on smoking cessation  □ Referred to smoking cessation clinic  □ Counselled on alcohol cessation  □ Referred for alcohol addiction |
| Environmental Health: | Do you have any pets? □ Cats □ Rodents □ Exotic Animals □Dogs □ No  Do you or your partner work with □ Pesticides □ Cleaning fluids □ Chemicals □ Paint □ Lead | □Counselled on limited exposure |
| Emotional Support: | Do you have emotional support at home? □ YES □ NO  Is help available from relatives or friends?  □ YES □ NO  Are you in a stable relationship? □ YES □ NO  Do you feel safe at home? □ YES □ NO  Are you physically threatened? □ YES □ NO  Do you feel good about yourself? □ YES □ NO  Have the following been diagnosed with depression? □ You □ Your family  □ Your partner □ None | □ Counselled on domestic abuse/IPV  □ Referred to social worker  □ Referred to psychiatrist |

| Additional Partner’s History | Age: Ethnicity:  Weight/kg: Waist Circumference/inches:  Height/m:  BMI (BSG chart):  Do they smoke cigarettes or use tobacco products? □ Yes □ No  If so, how many pack years? _____________  Are they exposed to secondhand smoke? □ Yes □ No  Do they drink alcohol? □ Yes □ No  If so, how much, how often: unit/d_____________  Do they use or have used drugs?  □ Cocaine □ Marijuana □ No □ Other __________  Do they use saunas or hot tubs?  Is their diet high in fat? □ Yes □ No  Is their diet high in processed foods? □ Yes □ No  How many glasses of the following do you they every day?  Tea Water  Coffee Milk  Cola Other  Do they exercise? □ Yes □ No  How many minutes per day/days per week?  □ <120 □ 120-150 □>150  Intensity: □ Mild □Moderate □ Vigorous | □ Requested to counsel partner |
| --- | --- | --- |

Investigations:

Presence of Acanthosis Nigricans: □ Yes □No

Grade of Acanthosis Nigricans (0-4): Texture of Acanthosis Nigricans (0-3)

Blood Pressure: Random Capillary Glucose CBC:

HbA1c:

CAN U add the presence and grade of acanthosis nigricans here!!

Reproductive Life Plan:
